# Co-creation and Preliminary Evaluation of Intergeneration Dialogue Board Game for Healthy Aging

**DOI:** 10.1093/geroni/igaf122.3240

**Published:** 2025-12-31

**Authors:** Eliza Lai-Yi Wong, Clement Cheuk-Wai Ng, Carol Ka-Po Wong, Shirley Shuk-Kuen Lui, Eng-Kiong Yeoh

**Affiliations:** The Chinese University of Hong Kong, Hong Kong, China (People’s Republic); The Chinese University of Hong Kong, Hong Kong, China (People’s Republic); The Chinese University of Hong Kong, Hong Kong, China (People’s Republic); The Chinese University of Hong Kong, Hong Kong, China (People’s Republic); The Chinese University of Hong Kong, Hong Kong, China (People’s Republic)

## Abstract

Social isolation is regarded as a priority global health issue, especially among older adults, while intergenerational communication could be a potential mitigating factor to alleviate social isolation by strengthening social connections and interactions between generations. The board game “Tackling Social Isolation: Dialogue Game on Healthy Growing and Aging”, which was developed by an expert panel comprising of a nurse, a social worker and a public health worker, covers 77 dialogue cards under six themes: ‘Grow-up Experience’, ‘Daily Life Encounters’, ‘Interpersonal Relationship’, ‘Health Literacy’, ‘Digital Literacy’, and ‘Action and Body Movement’. A total of 68 participants were recruited based on their age group as youth (18-39), middle-aged (40-64), and older adults (≥65) to enhance the intergenerational mix for the game dynamics. A pre-post intervention survey was conducted in 19 games to evaluate the board game experience. Results showed that most respondents agreed or strongly agreed that the board game could enhance their intergeneration communication (97.0%) and health knowledge (95.6%) as well as feeling better/much better (80.9%) in ‘able to cope with life’ post-intervention. The paired t-test results revealed that participants improved significantly on intergeneration communication, communication competence, and health literacy (p < 0.05). Focusing on the older adults (n = 31), medium effect sizes were observed in intergeneration communication (0.639) and health literacy (0.572), while small effect size was revealed in health literacy (0.402). This gamified intervention contributes to fostering engagement of all generations and improving social connection.

